# Prevalence and risk factors of gastroesophageal reflux disease in Iran: A cross-sectional analysis from the PERSIAN cohort

**DOI:** 10.1371/journal.pone.0306223

**Published:** 2024-07-11

**Authors:** Anahita Sadeghi, Paria Boustani, Ali Mehrpour, Ali Ali Asgari, Maryam Sharafkhah, Abbas Yazdanbod, Mohammad Hossein Somi, Azim Nejatizadeh, Farhad Moradpour, Mehdi Rezaeian, Fariborz Mansour-Ghanaei, Arman Shahriari, Mohammad Reza Fattahi, Behrooz Hamzeh, Seyed Vahid Hosseini, Mahmood Kahnooji, Ali Gohari, Mohammadreza Khosravifarsani, Hossein Azadeh, Mohammad Reza Pashaei, Eshagh Moradi Sheibani, Hossein Fallahzadeh, Alireza Bakhshipour, Hossein Poustchi, Reza Malekzadeh

**Affiliations:** 1 Digestive Diseases Research Center, Digestive Diseases Research Institute, Tehran University of Medical Sciences, Tehran, Iran; 2 Department of Internal Medicine, Tehran University of Medical Sciences, Tehran, Iran; 3 Digestive Disease Research Center, Ardabil University of Medical Sciences, Ardabil, Iran; 4 Liver and Gastrointestinal Diseases Research Center, Tabriz University of Medical Sciences, Tabriz, Iran; 5 Molecular Medicine Research Center, Hormozgan University of Medical Sciences, Bandar Abbas, Iran; 6 Social Determinants of Health Research Center, Research Institute for Health Development, Kurdistan University of Medical Sciences, Sanandaj, Iran; 7 Noncommunicable Diseases Research Center, Fasa University of Medical Sciences, Fasa, Iran; 8 Gastrointestinal and Liver Diseases Research Center, Guilan University of Medical Sciences, Rasht, Iran; 9 Alimentary Tract Research Center, Department of Internal Medicine, School of Medicine, Ahvaz Jundishapur University of Medical Sciences, Ahvaz, Iran; 10 Gastroenterohepatology Research Center, Shiraz University of Medical Sciences, Shiraz, Iran; 11 Research Center for Environmental Determinants of Health (RCEDH), Nutritional Sciences Department, Kermanshah University of Medical Sciences, Kermanshah, Iran; 12 Colorectal Research Center, Shiraz University of Medical Sciences, Shiraz, Iran; 13 Non-Communicable Diseases Research Center, Rafsanjan University of Medical Sciences, Rafsanjan, Iran; 14 Department of Biochemistry and Nutrition, School of Medicine, Sabzevar University of Medical Sciences, Sabzevar, Iran; 15 Department of Internal Medicine, School of Medicine, Hajar Hospital, Shahrekord University of Medical Sciences, Shahrekord, Iran; 16 Rheumatology Division, Department of Internal Medicine, Orthopedic Research Center, Mazandaran University of Medical Sciences, Sari, Iran; 17 Patient Safety Research Center, Urmia University of Medical Sciences, Urmia, Iran; 18 Department of Internal Medicine, School of Medicine, Yasuj University of Medical Sciences, Yasuj, Iran; 19 Research Center for Healthcare Data Modeling, Department of Biostatistics and Epidemiology, Shahid Sadoughi University of Medical Sciences, Yazd, Iran; 20 Research Institute of Cellular and Molecular Sciences in Infectious Diseases, Zahedan University of Medical Sciences, Zahedan, Iran; Kyung Hee University School of Medicine, REPUBLIC OF KOREA

## Abstract

**Background:**

This study assessed the prevalence of gastroesophageal reflux disease (GERD) in a general adult population in Iran. The association between GERD and various factors was also evaluated.

**Methods:**

We performed a cross-sectional study on 163,018 individuals aged over 35 who were enrolled in the PERSIAN cohort. GERD was defined as the occurrence of heartburn and/or regurgitation symptoms at least several days a month. Survey design analysis for pooled data was performed and multiple regression analysis was conducted to determine the independent risk factors for GERD.

**Results:**

The prevalence of GERD in our study was estimated at 21.86% (95% confidence interval:17.4%-36.4%). The mean age of the participants was 49.84 years±9.25 (35–70) and 44.75% of the participants were male. Symptoms of heartburn and regurgitation were reported in 18.65% (n: 29,170) and 6.06% (n: 9,717) of participants, respectively. In the multivariate analysis, several factors were found to be associated with a higher prevalence of GERD: female sex, age >50, current smoking, opium use, weekly consumption of fried foods, frequent consumption of hot tea, less than 6 hours of sleep per night, psychiatric disorders, usage of NSAIDs, and poor oral hygiene, were associated with a higher prevalence of GERD. Conversely, higher education levels and average physical activity were found to be less commonly associated with GERD.

**Conclusion:**

We found a relatively high prevalence of GERD (21.86%) in this population-based study in Iran. By identifying modifiable risk factors, this research offers opportunities for targeted interventions and lifestyle modifications to reduce the burden of GERD.

## Introduction

Gastroesophageal reflux disease (GERD) is one of the most common gastrointestinal disorders, characterized by the reflux of gastric contents into the esophagus. It has a high burden of disease and can reduce the quality of life of patients. GERD can present with typical symptoms such as heartburn and regurgitation, or with extra-esophageal symptoms such as cough, asthma, and laryngitis [1–3]. The global prevalence, incidence, and disability-adjusted life years (DALYs) of GERD have increased consistently from 1990 to 2019, reaching 783.95 million cases and 6.03 million DALYs in 2019. Population growth and aging are the main contributors to this trend [4–6].

The prevalence of GERD varies widely across different regions and populations, ranging from 2.5% to 52.1%. This reflects the influence of genetic, ethnic, lifestyle, and cultural factors on the development and manifestation of GERD. A systematic review estimated the prevalence of GERD as 18.1%–27.8% in North America, 8.8%–25.9% in Europe, 2.5%–7.8% in East Asia, 8.7%–33.1% in the Middle East, 11.6% in Australia and 23.0% in South America. East Asia seems to be less affected by GERD with a prevalence of lower than 10% in most of the studies. The studies estimating the prevalence of GERD in the Middle East are mostly in Iran reporting a wide range of 8.7% to 33.1%. Differences in the definition of GERD or study population (for example, distinct racial subgroups (may be a reason for this heterogeneity [7, 8].

Several risk factors have been identified for GERD, including genetic predisposition, socioeconomic status, dietary habits, body mass index (BMI), physical activity, pregnancy, age, sex, tea/coffee consumption, smoking, alcohol consumption, use of nonsteroidal anti-inflammatory drugs (NSAIDs), psychological stress and geographic differences [9–12].

To address the rising burden of GERD and the scarcity of epidemiological data in the Middle East, it is essential to estimate the prevalence of GERD and determine its risk factors in different populations of Iran, representing diverse ethnicities, regions, and cultures. This would help to establish appropriate management strategies and reduce the impact and costs of the disease [13, 14]. For this purpose, we conducted a nationwide study using data from the Prospective Epidemiological Research Studies in Iran (the PERSIAN Cohort Study) [15], the largest population-based cohort study in Iran that covers most provinces and major ethnic groups. This study aimed to investigate the prevalence and risk factors of GERD in a large cohort study in Iran, providing valuable insights into the epidemiology of GERD. The findings of this study can inform the development of public health strategies and clinical guidelines for the management of GERD, thereby improving patient outcomes and healthcare policy.

## Materials and method

A cross-sectional study was conducted to assess the prevalence and risk factors of GERD in Iran, using data from the PERSIAN cohort study, a nationwide project launched by the Ministry of Health and Medical Education in 2014 to evaluate noncommunicable diseases in Iran. The PERSIAN cohort study enrolled 163,770 individuals aged 35–70 years from 18 different geographic regions of Iran [15].

Data from the PERSIAN cohort study were accessed on 3^rd^ March 2023. Data were extracted from the PERSIAN cohort study using identification codes of all available variables. The original questionnaire in the PERSIAN cohort study asked about the frequency of heartburn and/or regurgitation symptoms, ranging from several times a day to several times a month. Since GERD is usually defined as having heartburn and/or regurgitation symptoms at least once a week, we included patients who reported having symptoms several times a month as well, to capture those who had symptoms only once a week. GERD was defined as the occurrence of heartburn and/or regurgitation symptoms at least several times a month, and severe GERD was defined as daily symptoms reported by the patient.

Data on age, sex, education level, marital status, area of domicile (urban/rural), wealth score, physical activity, medical history (depression, psychiatric disorder), habitual history (alcohol, opium, hookah, and smoking habits), fried food and smoked food consumption, tea consumption, hot soup and hot tea consumption, NSAIDs/Proton Pump Inhibitors (PPIs)/H2 Blockers intake, non-gas fuel exposure, sleep duration, water source, oral hygiene, other confounding symptoms (bloating, constipation, cough, shortness of breath) were all obtained using data from the PERSIAN cohort study [15]. BMI was calculated following the US National Institutes of Health protocols using the measured height (in cm) and weight (in kg). The physical activity level of participants was quantified using metabolic equivalents (METs) for 24-hour activities and categorized into three groups based on tertiles of the scores: low, moderate, and high. The wealth score index was calculated using multiple correspondence analyses on household wealth based on access or ownership of assets or amenities and was also divided into tertiles. Regarding the consumption of hot tea and hot soup, participants were asked about their preference for the temperature of these beverages and categorized into two groups: hot and not hot (including lukewarm, cold, or no consumption).

The primary outcome of this study was to determine the prevalence of GERD in the PERSIAN cohort. The secondary outcomes were determining the prevalence of GERD in different regions of Iran and evaluating various risk factors associated with GERD.

### Statistical analysis

In this study, we employed a complex survey design to derive summary measures, considering the use of cluster sampling. The probability of being selected in the survey was calculated based on data of the national census in 2016 and inverse probability was used as sampling weights for the analysis. We estimated the sex- and age-standardized prevalence of GERD in the total population and subgroups, using the 2016 national census as the standard population. We employed logistic regression models to assess the relationship between GERD and various risk factors [16]. Initially, we obtained age- and sex-adjusted odds ratios (ORs) along with 95% confidence intervals (CIs) for each variable. Subsequently, we included all variables with a p-value less than 0.2 in a multiple logistic model, and through backward model selection, we derived the final model. All analyses were performed using Stata software (version 14.1) (Stata Corp, College Station, TX, USA).

### Ethics statement

This study was conducted in accordance with the Declaration of Helsinki and ethical approval for this study was obtained from the Research Ethics Committees of Tehran University of Medical Sciences (IR.TUMS.DDRI.REC.1400.021). Informed written consent was obtained from all participants in the PERSIAN cohort study. The authors did not have access to information that could identify individual participants during or after data collection and all data were fully anonymized.

## Results

**Fig 1:**
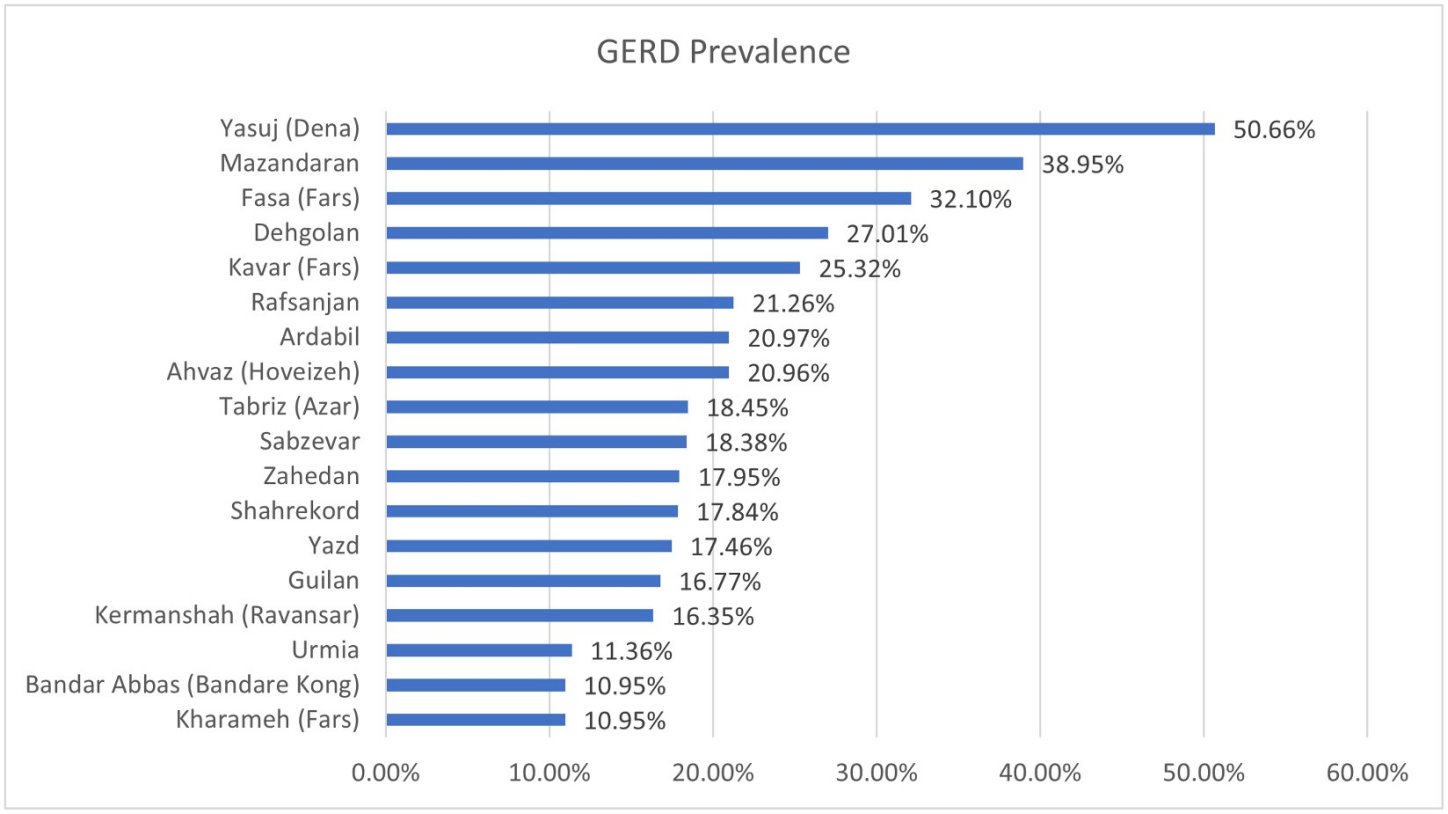
Prevalence of GERD in different cities of Iran.

Out of the 163,770 participants in the PERSIAN cohort, 752 (0.46%) individuals were excluded from the analysis due to missing data on the frequency of heartburn and/or regurgitation symptoms in the questionnaire. A total of 163,018 participants were included in the final analysis. The mean age of the participants was 49.84 years±9.25 (35–70) and 44.75% of the participants were male. Symptoms of heartburn and regurgitation were reported in 18.65% (n: 29,170) and 6.06% (n: 9,717) of participants, respectively. The prevalence of GERD in our study was estimated at 21.86% (95% confidence interval (CI):17.4%-36.4%), with 35.46% (n:11,916) mild, 37.58% (n: 12,271) moderate, and 26.96% (n: 10,247) severe cases. The prevalence of GERD in different cities of Iran is displayed in Fig 1.

### Univariate analysis

Table 1 presents the lifestyle and sociodemographic characteristics of participants and their association with GERD after adjusting for age and sex. The results revealed that age >50, female sex, low wealth score, smoking, opium use, weekly consumption of fried food, hot tea consumption, less than 6 hours of sleep a night, intake of NSAIDs, poor oral hygiene (brushing less than once a day or having dentures), and psychiatric disorders especially depression are considered risk factors for GERD. High education levels and average physical activity are considered protective factors. Initially, a crude association was observed between BMI and GERD in individuals with a BMI of 18.5–29.9 kg/m^2^ (OR:1.2; 95% CI: 0.9–1.5; p-value: 0.255), BMI of 30–40 kg/m^2^ (OR:1.3; 95% CI: 1.0–1.8; p-value: 0.038), and BMI >40 kg/m^2^ (OR:1.6; 95% CI: 1.2–2.3; p-value: 0.003) compared to individuals with a BMI <18.5 kg/m^2^. However, after adjusting for sex and age, the observed association no longer remained statistically significant.

**Table 1:** Lifestyle and sociodemographic characteristics of participants and their association with GERD.

	GERD Cases/ Total	GERD Prevalence^1^ (95% CI)	Odds Ratio^2^ (95% CI)	P-value
**Age group (years)**				
35–50 yrs	18,813/92,463	20.9 (16.9–25.0)	Ref.	
>50 yrs	15,621/70,555	23.4 (18.1–28.8)	1.15 (1.04–1.27)	0.007
**Sex**				
Male	12,323/72,943	18.01 (14.0–22.1)	Ref.	
Female	22,111/90,075	25.83 (20.8–30.8)	1.58 (1.50–1.68)	<0.001
**BMI (kg/m** ^**2**^ **)**				
<18.5	548/ 2,962	18.74 (15.0–22.5)	Ref.	
18.5–29.9	21,738/ 108,281	21.1 (16.3–25.9)	1.06 (0.82–1.38)	0.652
30–40	11,193/ 48,439	23.56 (19.2–27.8)	1.09 (0.83–1.44)	0.519
>40	748/ 2,694	27.51 (22.3–32.6)	1.24 (0.89–1.74)	0.185
**Education level**				
Low (illiterate)	8,606 / 33,592	28.48 (20.10–36.86)	Ref.	
1–5 Academic yrs.	11,378/ 51,831	23.68 (18.05–29.31)	0.82 (0.66–1.02)	0.079
5–12 Academic yrs.	11,233/ 58,105	20.23 (16.55–23.91)	0.73 (0.51–1.03)	0.072
High (>12 academic yrs.)	3,217 /19,490	16.68 (12.85–20.51)	0.59 (0.36–0.97)	0.037
**Marital status**				
Married	30,826 /148,426	21.51 (17.01–26.02)	Ref.	
Unmarried (single/ divorced/widowed)	3,608/ 14,592	26.14 (21.73–30.56)	1.06 (0.97–1.16)	0.164
**Area of domicile**				
Urban	24,595/ 115,243	21.02 (17.48–24.57)	Ref.	
Rural	9,839/ 47,775	24.73 (9.40–40.05)	1.22 (0.52–2.9)	0.633
**Wealth score**				
Low	12,907/ 55,732	26.98 (18.75–35.22)	1.47 (1.0–2.15)	0.049
Average	11,126/ 52,980	21.22 (17.39–25.04)	1.12 (0.99–1.28)	0.066
High	10,337/ 53,895	18.88 (15.56–22.20)	Ref.	
**Physical activity**				
Low	11,718/ 54,214	21.91 (18.99–24.84)	Ref.	
Average	11,588/ 54,186	21.23 (17.76–24.71)	0.88 (0.80–0.97)	0.013
High	11,052/ 54,144	22.48 (14.37–30.59)	1.07 (0.71–1.62)	0.733
**Smoking habits**				
Non- smoker	27,367/ 127,503	22.20 (17.52–26.88)	Ref.	
Ex-smoker	2,437/ 12,499	19.68 (15.40–23.96)	1.20 (1.08–1.33)	0.001
Current smoker	4,607/ 22,947	21.27 (17.35–25.19)	1.37 (1.22–1.54)	<0.001
**Hookah**				
No	31,409/ 150,175	21.89 (17.15–26.64)	Ref.	
Yes	2,996/ 12,758	21.50 (18.39–24.60)	1.14 (0.92–1.4)	0.227
**Opium**				
No	30,844/ 146,455	21.74 (17.13–26.35)	Ref.	
Yes	3,566/ 16,492	22.72 (18.45–27.0)	1.38 (1.16–1.63)	0.001
**Alcohol**				
No	32,299/ 152,498	21.96 (17.28–26.64)	Ref.	
Yes	2,110/ 10,446	20.57 (17.14–24.0)	1.22 (1.05–1.28)	0.106
**fried food (weekly consumption)**				
No	5,159/26,133	18.08 (15.34–20.82)	Ref.	
Yes	29,178/136,231	22.89 (17.92–27.81)	1.40 (1.10–1.81)	0.011
**Smoked Food**				
Never	28,787 /135,314	22.24 (17.07–27.42)	Ref.	
Ever	5,547/27,037	20.02 (19.11–21.31)	0.89 (0.68–1.18)	0.406
**Hot Tea Consumption**				
No	23,177/ 11,747	21.67 (17.28–26.06)	Ref.	
Yes	11,157/ 50,612	22.54 (17.78–27.28)	1.09 (1.04–1.14)	0.001
**Hot Soup**				
No	25,518/ 122,186	21.80 (17.24–26.36)	Ref.	
Yes	8,815/ 40,172	22.35 (18.00–26.72)	1.05 (1.0–1.11)	0.054
**Tea**				
Low (1–2 cups)/none	13,385/62,787	22.32 (18.2–26.4)	Ref.	
Moderate/High	20,970/99,707	21.68 (16.8–26.5)	1.02 (0.91–1.14)	0.735
**Non-gas fuel exposure (>5yr)**				
No	14,008 /70,075	19.73 (17.19–22.26)	Ref.	
Yes	20,426 /92,943	24.14 (16.78–31.50)	1.31 (0.89–1.91)	0.163
**Sleep amount**				
<6 hour	5,454 /23,668	24.21 (18.49–29.92)	1.17 (1.05–1.30)	0.005
6–8	21,510 /105,057	21.32 (16.66–25.98)	Ref.	
>8	7,470 /34,293	21.89 (18.19–25.59)	0.97 (0.85–1.09)	0.592
**Brushing (Daily)**				
Yes	13,176/ 66,889	19.92 (16.32–23.51)	Ref	
No	13,324/ 62,671	22.40 (16.83–28.0)	1.28 (1.05–1.57)	0.018
Has dentures	7,921/ 33,395	24.88 (19.25–30.52)	1.32 (1.11–1.58)	0.003
**Water Source** *				
Unhealthy	2,991 /12,734	32.04 (12.72–51.35)	0.55 (0.23–1.30)	0.167
Healthy	31,443 /150,284	20.76 (17.38–24.14)	Ref.	
**NSAID (weekly)**				
No	28,859 /142,152	21.04 (16.54–25.54)	Ref.	
Yes	5,575 /20,866	27.67 (22.71–32.63)	1.37 (1.19–1.58)	<0.001
**Depression** *				
No	28,957 /146,177	20.66 (15.76–25.56)	Ref.	
Yes	5,469 /16,810	31.55 (28.49–34.61)	1.59 (1.28–1.96)	<0.001
**Psychiatric Disorder** *				
No	31,265 /153,527	21.10 (16.69–25.52)	Ref.	
Yes	3,161 /9,460	34.01 (27.70–40.31)	1.80 (1.58–2.05)	<0.001

^1^ Sex- and age-standardized prevalence

^2^ Adjusted for sex and age

* Data on the water source, depression, and psychiatric disorders were collected through self-reporting. Participants were asked if they were diagnosed with depression or psychiatric disorders by a physician.

### Multivariate analysis

Table 2 presents the multivariate analysis of factors associated with GERD. Factors associated with GERD in the univariate analysis were included in the multivariate analysis. Age >50, female sex, current smoking, opium use, weekly consumption of fried food, hot tea consumption, less than 6 hours of sleep a night, intake of NSAIDs, poor oral hygiene (brushing less than once a day or having dentures), and psychiatric disorders especially depression remained risk factors for GERD in the multivariate analysis. High education levels and average physical activity were associated with a lower risk of GERD.

**Table 2:** Multivariate analysis of factors associated with GERD.

	Odds ratio (95% CI)	P-value
**Age group (years)**		
35–50	Ref.	
>50	0.96 (0.85–1.08)	0.489
**Sex**		
Male	Ref.	
Female	1.64 (1.50–1.78)	<0.001
**BMI (kg/m2)**		
<18.5	Ref.	
18.5–29.9	1.20 (0.95–1.50)	0.118
30–40	1.22 (0.95–1.56)	0.113
>40	1.31 (0.96–1.80)	0.087
**Education level**		
Low (illiterate)	Ref.	
1–5 Academic yrs.	0.81 (0.68–0.97)	0.022
5–12 Academic yrs.	0.74 (0.57–0.96)	0.026
High (>12 academic yrs.)	0.65 (0.46–0.93)	0.020
**Physical activity**		
Low	Ref.	
Average	0.91 (0.83–0.99)	0.026
High	1.07 (0.76–1.51)	0.694
**Smoking habits**		
Non- smoker	Ref.	
Ex-smoker	1.09 (0.99–1.20)	0.089
Current smoker	1.21 (1.07–1.37)	0.004
**Opium**		
No	Ref.	
Yes	1.17 (1.02–1.34)	0.026
**fried food (weekly consumption)**		
No	Ref.	
Yes	1.44 (1.11–1.87)	0.007
**Hot Tea Consumption**		
No	Ref.	
Yes	1.05 (1.00–1.11)	0.040
**Sleep amount**		
<6 hour	1.13 (1.02–1.24)	0.018
6–8	Ref.	
>8	0.94 (0.86–1.03)	0.162
**Brushing (At least once Daily)**		
Yes	Ref	
No	1.18 (1.02–1.37)	0.023
Has dentures	1.17 (1.02–1.33)	0.026
**NSAID (weekly)**		
No	Ref.	
Yes	1.31 (1.18–1.45)	<0.001
**Depression**		
No	Ref.	
Yes	1.53 (1.32–1.78)	<0.001
**Psychiatric Disorder**		
No	Ref.	
Yes	1.67 (1.49–1.88)	<0.001

The current study investigated potential associations between GERD and other accompanying gastrointestinal symptoms, such as bloating and constipation, as well as extra-gastrointestinal symptoms, such as cough and shortness of breath. The prevalence of bloating and constipation was noted to be considerably higher in individuals with GERD [%63.78 (95% CI:58.9–68.7) and %7.59 (95% CI:6.2–9.0)], in comparison to those without GERD [%37.28 (95% CI:32.8–41.7) and %4.15 (95% CI:3.5–4.8)]. Similarly, the occurrence of cough and shortness of breath was also notably higher in the GERD population [%16.64 (95% CI:12.3–21.0) and %8.52 (95% CI:6.4–10.6)], when compared to their counterparts without GERD [%10.87 (95% CI:8.05–13.7) and %3.91 (95% CI:2.9–4.9)].

The usage rate of PPI and H2 Blockers was %15.23 (95% CI:13.1–17.4) and %13.93 (95% CI:9.8–18.0) in the GERD population of our study.

## Discussion

In this cross-sectional study using data from the national PERSIAN cohort study, the prevalence of GERD was reported at 21.86% and age >50, female sex, current smoking, opium use, weekly consumption of fried food, hot tea consumption, less than 6 hours of sleep a night, weekly intake of NSAIDs, poor oral hygiene (brushing less than once a day or having dentures), and psychiatric disorders especially depression were identified as risk factors for GERD after controlling for confounding factors in the multivariate analysis.

The estimated prevalence of GERD in our study (21.86%) was similar to the prevalence in North America (19.55%) but exceeded the estimates for Europe (14.12%), Asia (12.92%) and Latin America and Caribbean (12.88%) [10]. Based on the results of a systematic review estimating the global prevalence of GERD including 96 studies [10], the prevalence of GERD in Iran was estimated at 18.43% which is consistent with our results. Previous studies in Iran evaluated the prevalence of GERD in limited regions [17–19]. Delavari et al. [20] conducted a qualitative systematic review of GERD prevalence in Iran, using all related published articles up to 2010. They found that there was a wide range of estimates of GERD prevalence in different studies.

They considered the Tehran study’s estimated prevalence of GERD (21.2%) to be the best approximation of GERD prevalence in Iran. Similarly, Fazel et al. [21] assessed articles containing estimates of GERD prevalence up to 2012. They found a wide range of estimates, from 1.9% to 52%, due to differences in the studies’ sampling and defining of GERD. These studies recommend large-scale comprehensive epidemiological studies to obtain more reliable results, as demonstrated in our study. Additionally, a meta-analysis of published studies up to 2019 by Karimian et al. [22] reported an overall prevalence of GERD symptoms at 43.07%, which was higher than our estimate. This could be because their overall estimate of GERD prevalence accounted for the monthly presentation of symptoms as well, leading to an overestimation.

Age over 50 was a risk factor for GERD in our study which is consistent with global trends, as shown in a meta-analysis that the pooled prevalence of GERD symptoms was higher in people over the age of 50, yet significant heterogeneity was observed among study results [8]. Another review demonstrated a higher prevalence of GERD in those aged 35–59 years than in those aged 18–34 or ≥60 years [10]. Age-related changes such as gastric dysmotility with delayed gastric emptying, incompetence of the LES, and impaired esophageal acid clearance due to disturbances of esophageal motility and saliva production in the elderly, are some of the reasons underlying the higher rate of GERD in older individuals [23]. Despite a large number of studies suggesting a positive correlation between increasing age and the prevalence of GERD [8, 24, 25], some studies indicate that younger age is associated with a higher risk of GERD, suggesting a shift in risk distribution toward younger age [26]. There is evidence that the proportion of younger individuals with GERD is increasing, particularly in the 30–39 years age group, indicating greater exposure to risk factors for GERD development in recent years [3]. As our study only included individuals over the age of 35, we may have missed a shift toward younger populations.

We found that GERD was more frequent in females. Consistently, pooled data from 50 previous studies around the world revealed that women suffered slightly more from GERD symptoms [10]. In addition, according to a meta-analysis, after adjusting data by geographical region of studies, the odds ratios among Middle Eastern women showed a modest increase [8]. The relaxing effect of female hormones on the LES has been suggested as a possible explanation for this disparity between males and females [27].

There seems to be a bidirectional association between GERD and sleep in the literature. A study evaluating the effect of sleep deficiency on esophageal acid exposure by Yamasaki et al. [28] showed increased esophageal acid exposure in sleep-deficient individuals in both healthy controls and patients with GERD. Furthermore, sleep disturbances are associated with the worsening of GERD symptoms or the occurrence of new-onset GERD symptoms [29, 30]. Sleep deficiency increases food cravings through hormonal alteration and the longer awake time can cause further food consumption. These changes could partly explain the association [28]. In concordance with previous studies, a significant association between GERD and sleeping less than 6 hours (OR: 1.17; 95% CI: 1.05–1.30; p-value:0.005) was observed in our study, compared to the average sleeping time of 6–8 hours. Moreover, GERD is correlated with new-onset insomnia, yet the significance of the risk of GERD as a consequence of sleep disturbances outweighs the opposite causation [29].

There also seems to be a bidirectional association between GERD and psychiatric disorders, especially depression. A case-control study using data from the Korean National Health Insurance Service- National Sample Cohort, showed higher ORs for depression in patients with GERD compared to the control group and higher ORs for GERD in the depression group [31]. Moreover, a recent systematic review exploring the association between anxiety/depression and GERD, suggests a bidirectional causal relationship between anxiety/depression and GERD [32]. Psychological factors might increase acid reflux by lowering the LES pressure, increasing gastric acid in the esophagus, and changing esophageal motility. Additionally, psychological factors exaggerate the sensation of esophageal stimulation and reflux symptoms by lowering the sensation threshold [31, 33]. In line with previous studies, we found GERD significantly associated with psychiatric disorders (OR:1.80; 95% CI:1.58–2.05; p-value <0.001) and depression (OR:1.59; 95% CI:1.28–1.96; p-value <0.001). However, in a recent bidirectional Mendelian randomization study, GERD significantly increased the risk of anxiety disorders and depression, yet anxiety or depression did not increase the risk of GERD reversely [34]. Increased inflammation levels and sleep disturbances caused by GERD are possible explanations contributing to the increased risk of anxiety disorders and depression.

Average physical activity was considered a protective factor for GERD in both univariate (OR:0.88 95% CI:0.80–0.97; p-value:0.013) and multivariate analysis. However, no association was found between high or low levels of physical activity and GERD. These results align with the findings of a previous study by Jan Bilski et al. [35] where moderate exercise showed beneficial effects on GI tract disorders including reflux esophagitis and high-intensity or prolonged endurance training had a negative impact. It has been suggested that running and intense exercise exacerbate GERD symptoms by increasing the percentage of transient lower esophageal sphincter relaxations, negatively impacting athletes’ performance [36, 37]. Nonetheless, regular physical activity and exercise offer countless health benefits. The current study indicates that moderate physical activity is beneficial in reducing GERD symptoms and should be recommended to GERD patients yet caution regarding the intensity and duration of exercise is required.

The univariate analysis revealed a significant positive association between current smoking (OR:1.37; 95% CI:1.22–1.54; p-value<0.001) and ex-smoking (OR:1.20; 95% CI:1.08–1.33; p-value: 0.001) with GERD. However, after controlling for confounding factors in multivariate analysis, only current smoking was significantly associated with GERD. These findings suggest that quitting smoking may have beneficial effects on GERD. It’s recommended that patients with GERD quit smoking to improve GERD and health-related quality of life [38].

The present study findings indicate that weekly use of NSAIDs is associated with an elevated risk of GERD (OR: 1.37; 95% CI: 1.19–1.58; p-value < 0.001). It is worth noting that NSAIDs are widely used, with an estimated 30 million individuals consuming these drugs daily [39]. A significant proportion of NSAID users—up to 40%—experience upper GI symptoms, with GERD being the most frequently reported [40]. In a large-scale study involving 6823 French adults, Ruszniewski et al. [41] similarly observed significantly higher rates of GERD symptoms among NSAID users relative to non-users (27% vs. 19%). Several prior studies have also established a positive association between NSAID use and the risk of developing GERD [42–44]. Moreover, NSAIDs have been implicated in increasing the likelihood of GERD relapse, with even monthly consumption of these drugs associated with a higher chance of recurrence [45]. The correlation between the use of NSAIDs and GERD underscores the significance of careful medication management and the evaluation of alternative approaches for pain management in individuals with a predisposition towards GERD.

A systematic review and meta-analysis examining the relationship between BMI and GERD including a comprehensive analysis of 20 studies, revealed a positive association between increasing BMI and GERD in studies conducted in the United States. However, the results of European studies exhibited considerable heterogeneity [46]. Furthermore, a large-scale study conducted among 10,545 women in England demonstrated a dose-dependent relationship between BMI and GERD symptoms [47]. The present study indicated 24% greater odds of GERD with a BMI>40 (OR: 1.24; 95% CI: 0.89–1.74; p-value: 0.185). However, this association did not reach statistical significance even after controlling for potential confounding factors through multivariate analysis (OR: 1.31; 95% CI: 0.50–0.12; p-value: 0.087). Interestingly, multiple studies conducted in the Iranian population have consistently reported no significant association between BMI and GERD, contrasting the findings observed in Western populations [48–51]. It is plausible to speculate that discrepancies in ethnicity, social factors, and lifestyle between Iran and the Western population may contribute to this discrepancy.

The study’s results are consistent with global trends and contribute to a better understanding of GERD’s prevalence and risk factors in Iran. Furthermore, it emphasizes the importance of large-scale epidemiological studies to obtain reliable data that can inform public health policies and clinical guidelines. To the best of our knowledge, it was the first study to evaluate GERD covering different geographical regions of Iran containing almost all ethnicities. Evaluating a wide range of lifestyle and environmental variables was another positive feature. This study has several limitations. One major limitation is its cross-sectional design, which prevents establishing causal relationships. To reduce bias resulting from linguistic or cultural differences, all cohort staff were local to each site and followed the same protocol. However, the consistency of the interviews may have been subject to interobserver variation. To minimize this, all interviewers received training at the same center. Routine assessments and monitoring were carried out to ensure data collection followed protocols. Another limitation is that the PERSIAN cohort, like other large population-based cohort studies, does not include a random sample of the Iranian population, limiting its representativeness. Additionally, data on water sources, depression, and psychiatric disorders were collected through self-reporting, which has its limitations. Self-reporting may be susceptible to biases such as recall bias and social desirability bias, which may have influenced the accuracy of the reported data. These biases should be considered when interpreting the results.

## Conclusions

In this cross-sectional study, utilizing data from the national PERSIAN cohort study, the prevalence of GERD in Iran was found to be 21.86%. Significant associations were identified between GERD and factors such as age over 50, female sex, current smoking, opium use, dietary habits, sleep patterns, oral hygiene, psychiatric disorders, and NSAID use. These findings underscore the multifactorial nature of GERD and the need for a comprehensive approach to its prevention and management. By identifying modifiable risk factors, this research offers opportunities for targeted interventions and lifestyle modifications to reduce the burden of GERD. Future research should focus on longitudinal studies to establish causal relationships between identified risk factors and GERD, explore the impact of GERD on quality of life, and evaluate the effectiveness of preventive measures and treatments.
